# Emergent weak home-range behaviour without spatial memory

**DOI:** 10.1098/rsos.160214

**Published:** 2016-06-29

**Authors:** Tomoko Sakiyama, Yukio-Pegio Gunji

**Affiliations:** 1 Department of Intelligent Mechanical Systems, Graduate School of Natural Science and Technology, Okayama University, Okayama, Japan; 2 Department of Intermedia Art and Science, School of Fundamental Science and Engineering, Waseda University, Tokyo, Japan

**Keywords:** home range, agent-based model, movement strategy, exploitation, exploration

## Abstract

Space-use problems have been well investigated. Spatial memory capacity is assumed in many home-range algorithms; however, actual living things do not always exploit spatial memory, and living entities can exhibit adaptive and flexible behaviour using simple cognitive capacity. We have developed an agent-based model wherein the agent uses only detected local regions and compares global efficiencies for a habitat search within its local conditions based on memorized information. Here, memorized information was acquired by scanning locally perceived environments rather than remembering resource locations. When memorized information matched to its current environments, the agent changed resource selection rules. As a result, the agent revisited previous resource sites while exploring new sites, which was demonstrating a weak home-range property.

## 1. Introduction

Various movement strategies have been studied, and such knowledge is useful for understanding the behavioural and cognitive processes of living systems [1–3]. Actual animals search within limited regions; therefore, space use for such animals has also been investigated extensively [2,4,5]. Space use for animals is strongly affected by environmental contexts, such as resource distributions, topography and the presence of other animals [6–9]. Two approaches are used for space-use problems. One is a statistical approach wherein space use is estimated by fitting statistical models to animal location data [5,10,11]. Although this method can illustrate realistic agent space use, it lacks the descriptions of processes wherein agents form a home range or habitat selection [2]. Thus, connections between the emergent global patterns and the cognitive or behavioural processes of agents are unclear in this method. The other approach is a mechanistic approach that attempts to construct the models of animal movements [4,9,12–14]. This method can capture animal movements to a degree; however, many studies assume that agents can use spatial memory or maps to remember previously visited positions [1,4,6,9,15,16]. These assumptions appear to help space-use or random walk models by allowing memory to converge to a home range [2]. However, not all living things exploit spatial memory [17]. Spatial knowledge is not required for agents that hold variable interpretations of exploration efficiencies triggered by local information. Such agents would exhibit adaptive behaviours for complex or unstable environments by modulating their behaviours without exploiting extravagant abilities [18–20]. This implies that an agent's decision-making or exploration strategies can be affected by interactions with the environment or the surrounding condition. Here, we show that agents that see limited local areas can identify a weak home range by changing their resource selecting strategies. Note that our models partly follow Arthur *et al.* [6] and Rhodes *et al.* [8], in which resource selection analysis related to a home range was developed by encouraging the agent to select nearby habitats based on its current position. It is reasonable to develop algorithms of resource selection strategies in which agents consider global exploration efficiencies by detecting limited local areas when they cannot use spatial memory. Here, memorized information is acquired by scanning locally perceived environments rather than remembering resource locations. The goal of this study is to investigate whether such a low-capacity agent can exhibit a somewhat weak home range during foraging and demonstrate the balance between exploitation (a home range) and exploration (visiting new sites) relating to movement strategy. In the proposed algorithm, the agent settles on a two-dimensional field in which sites associated with food sources are distributed randomly. The agent selects one site if it can detect appropriate sites locally based on resource selection rules. The resource selection rule is changed when the agent doubts the global exploration efficiency of the current rule. As discussed later, this event relies on the agent's uncertain scene memory. As a result, a weak home-range property emerges, which demonstrates both revisiting previous sites (cycles) and exploring new sites. A home range can be defined as an emergent circle in which agents revisit several previous sites [21]. This result can be related to the balance of exploitation and exploration.

## 2. Material and methods

### 2.1. Basic description

Here, the field is a 100.00 × 100.00 grid, and an initial agent position (50.00, 50.00) is set for each trial. One thousand randomly distributed sites are introduced to the field. In this algorithm, the agent selects local sites based on its rule. Here, the visual field of the agent has a radius of 4.00. If sites are located in the agent's visual field, it selects one site from the detected sites. We define the detected sites as local sites. The total number of local sites varies depending on the agent's position. Once one site is chosen by the agent, the agent chooses the other site from local sites.

We also define two selection rules. One is an exploitation rule wherein the agent selects the nearest site from its current position. In the exploitation rule, the agent attempts to conserve transportation and energy costs by effectively selecting the nearest sites. The other rule is an exploration rule wherein the agent selects a site furthest from the local sites. In the exploration rule, the agent attempts to search wide areas by selecting such sites. These rules are local rules for the agent; therefore, the agent cannot assess the global efficiencies of the rules, because we assume that the agent cannot use global cognitive maps. Thus, these rules are not necessarily in opposition when the agent considers global properties, even though they conflict locally. Note that each trial has 10 000 time steps.

These settings are the basic settings for all models described in the following sections.

### 2.2. Rule-change model

We set the exploitation rule as the default rule in this model. This is a natural condition for agents' movement strategy algorithms [6,8]. If the total number of local sites is zero, i.e. no sites are located in the agent's current visual field, the agent selects one direction at random (north, east, west or south) and updates its position with step size 1.

Roughly speaking, in this algorithm, if the agent understands or misunderstands its current position as an already visited site, the agent doubts the global efficiencies of its current rule and changes rules to achieve its global exploitations and search. This is described as follows.

**Figure 1.**
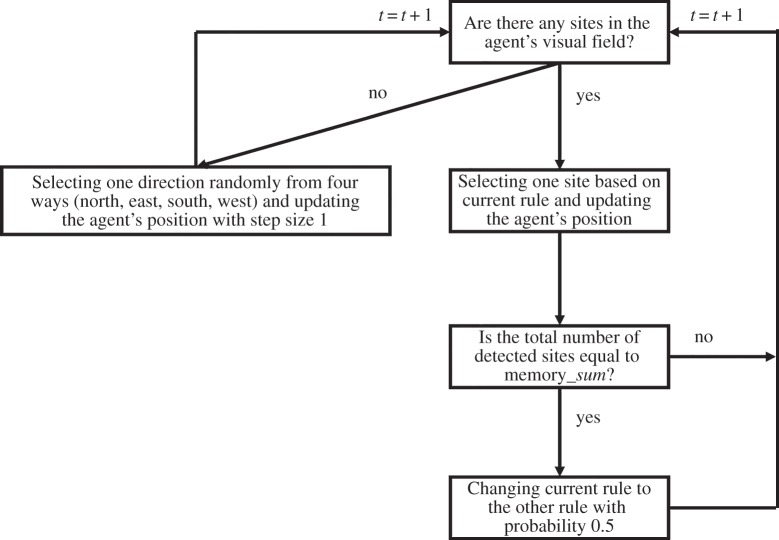
Rule-change algorithm flow chart (detected sites indicate local sites from the agent's current position).

In our algorithm, an agent perceives the total number of local sites in each time step. Memorized information for the agent is scene memory by scanning the total number of local sites perceived at its position [21]. The first time the agent detects local sites in each trial, it memorizes the total number of local sites at that location. We define that value as memory_*sum*. If the total number of local sites of the current position is equal to the agent's memorized value (memory_*sum*), the agent replaces the current rule with the other rule with probability 0.5. We set the time length of memory storage to *θ*. Therefore, if *θ* time step lengths have passed as the agent updated memory_*sum*, that value will be re-updated and replaced with the total number of local sites at the agent's current position. For example, if *θ* = 10, after scanning a certain landscape scene (memorizing the value ‘memory_*sum*’), the agent can compare current local views with memory_*sum* for 10 time steps. After 10 time steps from the time the agent retains memory_*sum*, the agent forgets memory_*sum* and re-updates that value when the agent perceives local sites (figure 1; electronic supplementary material). Matching its current scene to a memorized one induces the agent to understand that it returns to previously visited sites. Note that the agent does not always actually return to previously visited sites when it replaces its selection rule with the other rule. The agent must judge whether its current positions are new sites or not using memorized information because the agent cannot use spatial memory. Thus, in order to visit new sites, the agent interprets the current rule as unfavourable when it replaces its rule with the other rule. Here, we set *θ* = 5.

### 2.3. Control models: without spatial memory

In addition, we have developed three control algorithms in which we forced agents to select the nearest site (fixed exploitation), the most distant site (fixed exploration) or one random site (random choice) from the local sites. Thus, in these algorithms, the agents do not change their site-selection conditions until each trial terminates.

As discussed in the following section, the agent in the rule-change model can visit more sites than in the two fixed control models (fixed exploitation and fixed exploration). It also demonstrates power-law tailed distributions, which is never achieved in the random-choice control model.

### 2.4. Control model: with spatial memory

We have developed a spatial memory-based algorithm in which the agent could store locations of detected sites. Our model is based on recent studies [14,22]. The agent can use two different memories. One is the reference memory, which is used by the agent to revisit previously visited sites. Memorized information will be forgotten if the agent does not return to that site within a duration *θ*_reference_. The other memory is the working memory, which is used by the agent to avoid coming back to visited sites soon. Memorized information will be lasting *θ*_work_. Note that *θ*_work_ < *θ*_reference_. The agent returns to a visited site with a certain probability (*prob*) if the working memory is vanished while the reference memory is alive regarding that site. If there are other visited sites on equal terms with that site, the agent randomly selects one site from those sites. If the parameter *prob* is close to 0, the agent hesitates to return to previously visited sites. Contrary to that, the parameter *prob* is close to 1, the agent is attracted to previously visited sites. When the agent does not or cannot select any previously visited sites, the agent adopts the exploitation rule. The number of memorized locations of visited sites (*N*_locations) was set to 1 in order to compare the rule-change model with the spatial memory-based model (*N*_locations = 1). In the rule-change model, the agent is allowed to memorize only one snapshot of information. The parameter *θ*_reference_ and *θ*_work_ were set to 10 and 5, respectively, because we set the time length of memory storage *θ* = 5 in the rule-change model. We calculated total number of visited sites using both the rule-change model and the spatial memory-based model by setting the parameter *prob* = 0.20, 0.50, 0.80 (see the electronic supplementary material for details).

## 3. Results

Here, we discuss the results obtained with the rule-change model and compare the results with those of the control models. Then, we demonstrate how parameter settings influence the results.

**Figure 2.**
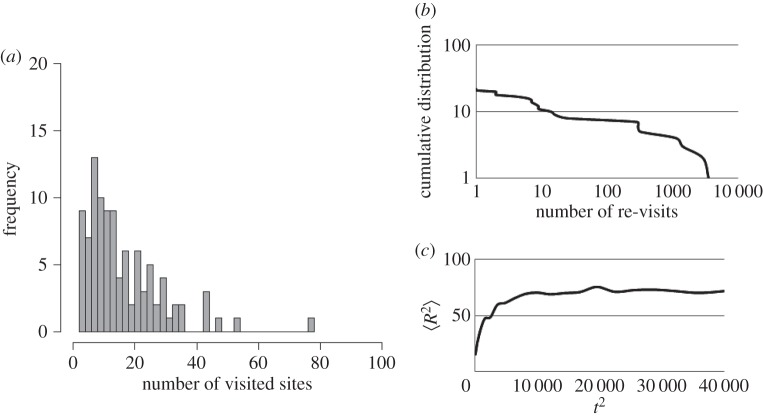
Results of rule-change model: (*a*) frequency of the number of visited sites in each trial (100 trials). For example, the frequency ‘15’ means that in 15 of the trials, the number of visited sites is between 6 and 8. (*b*) Cumulative distribution of the number of re-visits to each site. A few sites were visited thousands of times in this trial. Contrary to that, the point where the curve meets the *y*-axis means the number of sites visited a few times. (*c*) Relationship between the mean-squared displacements 〈*R*^2^〉 and the time squared *t*^2^ (100 trials).

Figure 2*a* illustrates the distributions of the total number of visited sites after each trial. These data were obtained by conducting 100 trials. It seems that the agent visits approximately less than 20 sites in each trial. As can be seen in figure 2*b*, which shows a power-law tailed distribution relative to the number of visits to each site after one trial, the agent appears to occasionally re-visit certain sites while visiting new sites (the number of data = 22, AIC weights of power-law = 1.00, *μ* = 1.31).

Figure S1 in the electronic supplementary material shows some examples of density of the agent's trajectories calculated in a single trial. The bin widths were set to 4.00 for each axis (*x* and *y*). The agent appears to search in a limited region in each trial. However, sometimes the centre of the search areas appears to shift (electronic supplementary material, figure S1*b*). After shifting the areas, the agents again search in a limited region. These typical properties are also shown in figure 2*c*, which shows the diffusion property of the agent. One hundred trials were conducted, and the average values are shown in figure 2*c* using a time step bin size of 10. After approximately 100 time steps, the mean-squared displacements are saturated. These results indicate that a weak home-range property which induces the agent to both revisit previous sites and explore new sites is achieved with this algorithm.

**Figure 3.**
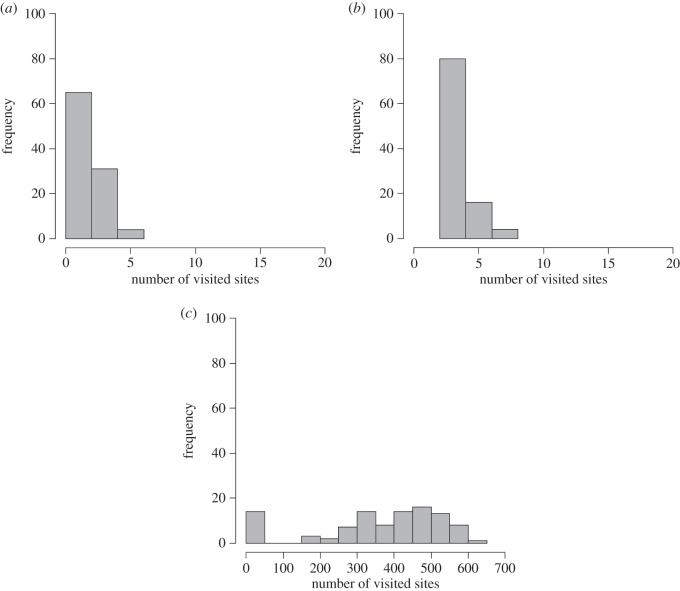
Frequency of the number of visited sites after each trial (100 trials) for the three control models: (*a*) Fixed-exploitation model, (*b*) fixed-exploration model and (*c*) random-choice model.

Next, we evaluated the total number of visits for each site after each trial using the three control algorithms. As seen in figure 3, the fixed-exploitation and fixed-exploration models cannot induce the agent to visit as many sites as with the rule-change model (rule-change model versus fixed-exploitation model: mean = 16.66 versus mean = 2.49, Mann–Whitney *U* test, *U* = 186.50, *p* < 1.0 × 10^−15^; rule-change model versus fixed-exploration model: mean = 16.66 versus mean = 3.91, Mann–Whitney *U* test, *U* = 627.50, *p* < 1.0 × 10^−15^). By contrast, the agent in the random-choice model can visit more sites than with the rule-change model (rule-change model versus random-choice model: mean = 16.66 versus mean = 361.30, Mann–Whitney *U* test, *U* = 191.50, *p* < 1.0 × 10^−15^). However, no power-law tailed distribution is achieved in that model relative to the number of visits to each site after a single trial. The random-choice model may not be able to demonstrate any critical behaviours (electronic supplementary material, figure S2*a*; the number of data = 498, AIC weights of power-law = 0.00, *λ* = 0.052). In addition, the mean-squared displacements are not saturated in this model. Figure S2*b* in the electronic supplementary demonstrates the result of mean-squared displacements obtained from 100 trials.

We then checked how parameter settings influence our results. We first replaced 1000 sites randomly distributed on the test field with 500 or 1500 sites. As a result, the number of local sites for the agent will change. Even though the densities of sites are modulated, the rule-change model is better than any of the fixed control models (electronic supplementary material, figure S3*a* and S3*b*; the number of sites = 500; rule-change model versus fixed-exploitation model: mean = 6.39 versus mean = 2.32, Mann–Whitney *U* test, *U* = 1111.00, *p* < 1.0 × 10^−15^; rule-change model versus fixed-exploration model: mean = 6.39 versus mean = 2.61, Mann–Whitney *U* test, *U* = 1410.00, *p* < 1.0 × 10^−15^; the number of sites = 1500; rule-change model versus fixed-exploitation model: mean = 15.50 versus mean = 3.00, Mann–Whitney *U* test, *U* = 8.00, *p* < 1.0 × 10^−15^; rule-change model versus fixed-exploration model: mean = 15.50 versus mean = 2.00, Mann–Whitney *U* test, *U* = 7.50, *p* < 1.0 × 10^−15^).

Figure S3*c*,*d* in the electronic supplementary material, figures show the distribution of the total number of visited sites obtained from 100 trials when *θ* was changed from 5.00 to 10.00 or 20.00, respectively. If the memory capacity of the agent increases, the agent visits fewer sites (*θ* = 5.00 (default) versus *θ* = 10.00): mean = 16.66 versus mean = 7.56, Mann–Whitney *U* test, *U* = 2294.50, *p* < 1.0 × 10^−10^). This behaviour appears to reach its peak at *θ* = 10.00 versus *θ* = 20.00: mean = 7.56 versus mean = 7.07, Mann–Whitney *U* test, *U* = 4915.00, *p* = 0.85, n.s. Thus, high memory capacity is not required for flexible agent behaviours.

Finally, we examined whether or not searching efficiencies of the rule-change model differed from spatial memory-based models. Therefore, we developed a model based on spatial memory-based models [14,22]. We compared the total number of visited sites using the rule-change model with those values of the spatial memory-based model. Figure S4 in the electronic supplementary material represents the averaged total number of visited sites obtained from 100 trials. Each trial lasted 200 time steps. The rule-change model is more effective than the spatial memory-based model (the rule-change versus the spatial memory-based (*prob* = 0.20): mean (10.95) versus mean (4.49), Mann–Whitney *U* test, *U* = 1443.5, *p* < 1.0 × 10^−15^; the rule-change versus the spatial memory-based (*prob* = 0.50): mean (10.95) versus mean (4.17), Mann–Whitney *U* test, *U* = 1217.5, *p* < 1.0 × 10^−15^; the rule-change versus the spatial memory-based (*prob* = 0.80): mean (10.95) versus mean (3.78), Mann–Whitney *U* test, *U* = 883.5, *p* < 1.0 × 10^−15^). Actually, we could obtain similar results between two models if the parameter *N*_locations (the number of memorized locations of visited sites) was changed to 5 from 1 (the rule-change versus the spatial memory-based (*prob* = 0.80, *N*_locations = 5): mean (10.95) versus mean (8.56), Mann–Whitney *U* test, *U* = 4349.5, *p* = 0.11, n.s.).

## 4. Discussion

The results clearly show that changing resource selection rules for local sites based on uncertain scene memory induces the agent to move within restricted regions while occasionally visiting new sites, which demonstrates a weak home range (emergence and breaking circuits). In the proposed algorithm, the agent changes from the resource selection rule to the other rule when it understands that it has returned to previously visited sites. The important contribution of the proposed model is that the agent does not always return to previously visited sites when the selection rule changes. Sometimes the agent misidentifies new locations as previously visited locations. This could be related to the emergence of the power-law tailed distributions relative to the number of revisited sites and the occasional shifts of the centre of restricted areas. When the agent's resource selection rule is fixed to one type, the agent cannot visit as many sites compared with the rule-change model. When the agent selects a local site randomly, no power-law distribution emerges relative to the number of revisited sites. This model appears to be specialized to wide exploration rather than moving within restricted regions. Interestingly, the agent in the rule-change model can visit more sites compared with the spatial memory-based model. In our situations, the agent appears to get pinned between several sites even it is not attracted to previously visited sites. Thus, rule-change strategy is a key point for achieving wide-range searching.

The proposed model is flexible with regard to changes in site density. In addition, a large memory capacity is not required to achieve effective searching, perhaps because the time until the agent departs a loop would increase with memory capacity.

With the proposed algorithm, the agent sometimes regards new locations as previously visited locations and, therefore, changes the rule selection procedure to maintain effective searching. In this case, the agent interprets the current rule as unfavourable. The agent cannot trace global trajectories because of limited visual ability and memory. Even though its actual movements demonstrate a wide search range, given the current rule, the agent occasionally understands that it has returned to previously visited locations.

Real living systems also modulate behaviours in various ways based on limited information [18–20]. The use of limited information will depend on the agents' experiences and environmental conditions [18,19]. Insects might store landscape views or visual landmarks as snapshots for their foraging [17,23]. In addition to that, they appear to associate food locations with visual landmarks [17]. Therefore, detecting each food site can be replaced with recognizing each visual landmark when agents can associate food with such landmarks. Detecting all the sites within a particular range of perception would be replaced with detecting current visual landscapes containing indistinguishable landmarks or visual cues scanned as snapshots. In that sense, it is not important in our model that sites recover because insects and larger animals might recognize whether or not food is available on that site after reaching that site [17]. Even if food is not available on that site, they tend to continue exploring new food locations [24]. This behaviour can be partly similar to choosing one site one after another in our model. Although there is little evidence whether or not real insects can change choosing strategy, our model might be compared with movement strategy of animals such as insects living in the environments over which indistinguishable visual cues are scattered. The proposed model allows agents with limited memory capacity to exhibit somewhat home-range behaviours and achieve the balance between exploitation and exploration. Getting pinned between several sites (exploitation) is effective for foraging because animals might visit a few sites reliably in a small region by returning to a certain area. However, in the long term, causing the animal to slowly wander around the whole terrain (exploration) and removing home-ranging behaviour are important to discover more profitable or new food locations. Thus, the balance between exploitation and exploration is exhibited as a weak home range [20].

## Supplementary Material

Title:Supporting file Description: Model description and supporting figures.
