# Supplementary material for: Emergent weak home-range behaviour without spatial memory
Source: R Soc Open Sci. 2016 Jun 29;3(6):160214. doi: 10.1098/rsos.160214 (PMC4929913; doi:10.1098/rsos.160214)
Supplement: Title:Supporting file Description: Model description and supporting figures. [file rsos160214supp1.docx]

**Rule-change model**

Followings are equations and/or pseudocode of *the rule-change model*.

On each trial, each value is initially set to followings.

*memory_sum* =0

*current position* = (50.00, 50.00)

*t* = 0.

At time *t*,

If *memory_sum* =0,

then the agent counts the total number of local sites at time *t* (*n*_t_).

if *n*_t_ > 0

then, memory_sum = *n*_t_

*memory_timespan* = *t*

and updates the agent’s position as following.

*current position* = *site*_k_

where *current position* is the agent’s current position and *site*_k_ is nearest site (exploitation rule) or farthest site (exploration rule) among {*site*_1_,*site*_2_,・・・, *site_n_*_t_}.

Here, {*site*_1_,*site*_2_,・・・, *site_n_*_t_} indicates the set of positions of local sites detected by the agent at time *t*.

*else if n*_t_ = 0

then, updates the agent’s position by selecting one direction at random (north, east, west, or south) and updates its position with step size 1.

Else if *memory_sum* > 0,

then the agent counts the total number of local sites at time *t* (*n*_t_)

if *n*_t_ > 0

then, updates the agent’s position as following.

*current position* = *site*_k_

where *current position* is the agent’s current position and *site*_k_ is nearest site (exploitation rule) or farthest site (exploration rule) among {*site*_1_,*site*_2_,・・・, *site_n_*_t_}.

Here, {*site*_1_,*site*_2_,・・・, *site_n_*_t_} indicates the set of positions of local sites detected by the agent at time *t*.

if (*t*- *memory_timespan)* < *θ*

then changes the agent’s rule from current rule to another rule with probability 0.5

else if (*t*- *memory_timespan)* >= *θ*

then, memory_sum = *n*_t_

*memory_timespan* = *t*.

*else if n*_t_ = 0

then, updates the agent’s position by selecting one direction at random (north, east, west, or south) and updates its position with step size 1.

Then *t*=*t*+1.

**Spatial memory-based model**

Followings are equations and/or pseudocode of *the spatial memory-based model*.

On each trial, each value is initially set to followings.

*θ*_refference_ =10,

*θ*_work_ = 5,

*current position* = (50.00, 50.00),

*t* = 0,

*T_site_*_1_=0, *T_site_*_2_ = 0, ・・・, *T_site_*_N_ =0,

where *N* represents the number of sites distributed on the field

At time *t*,

if there are sites satisfying following equation,

(*t*-*T*_site_*_k_*) <*θ*_refference_ && (*t*-*T*_site_*_k_*) >=*θ*_work_ && *T*_site_*_k_* > 0 && random_number*_t_* < *prob,*

(*k*=1, 2, ・・・ *N*)

where, *T*_site_*_k_* indicates the latest time at which the agent visited *site*_k_,

random_number*_t_* represents the random number on time *t*,

random_number*_t_* = [0.00, 1.00]

then, the agent randomly selects one site from above sites und updates the agent’s position and update *T_site_*_m_ = *t*,

where *site*_m_ is chosen site by the agent

else,

if *n*_t_ > 0

then, updates the agent’s position as following.

*current position* = *site*_k_

where *current position* is the agent’s current position and *site*_k_ is nearest site (exploitation rule) among {*site*_1_,*site*_2_,・・・, *site_n_*_t_}.

Here, {*site*_1_,*site*_2_,・・・, *site_n_*_t_} indicates the set of positions of local sites detected by the agent at time *t*.

and memorizes location of the agent’s current position and updates *T_site_*_k_ = *t*, if total number of memorized sites < *N*_locations

*else if n*_t_ = 0

then, updates the agent’s position by selecting one direction at random (north, east, west, or south) and updates its position with step size 1.

if (*t*-*T*_site_*_p_*) = *θ*_refference_ && *T*_site_*_p_* > 0, (*p*=1, 2, ・・・ *N*)

the agent forgets location of *site*_p_ and updates *T*_site_*_p_* = 0

Then *t*=*t*+1.


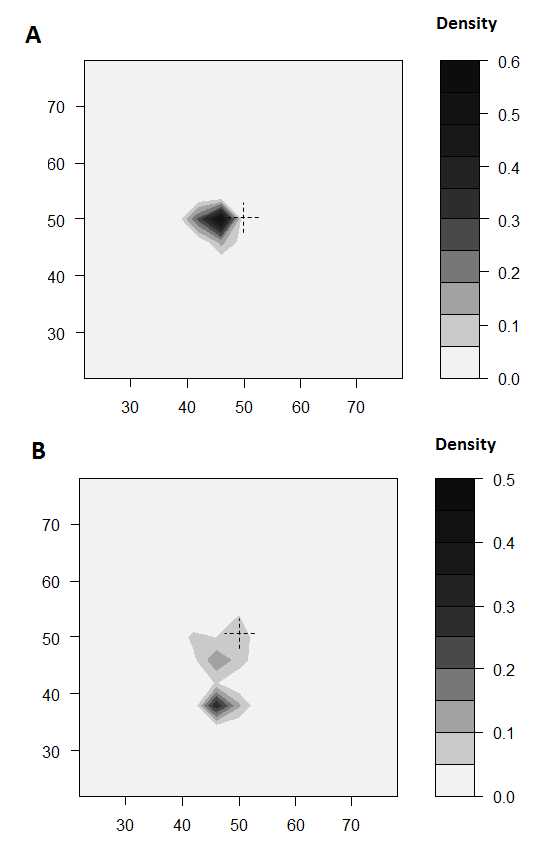


Figure S1


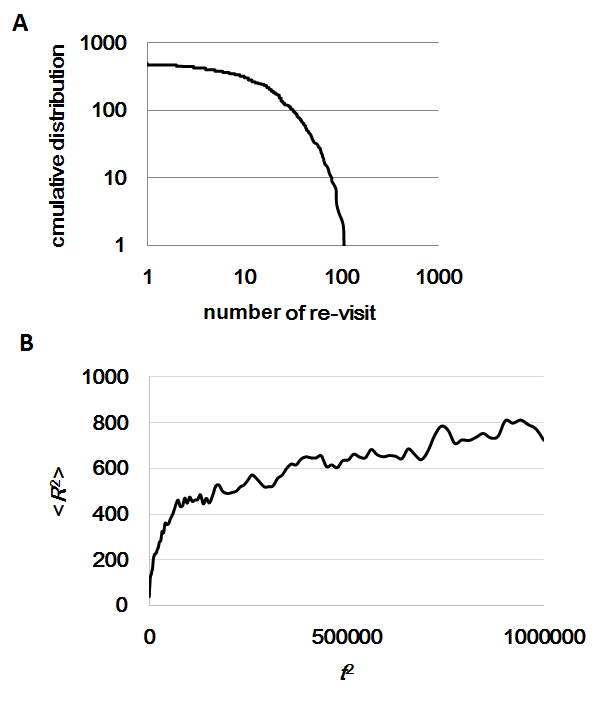


Figure S2


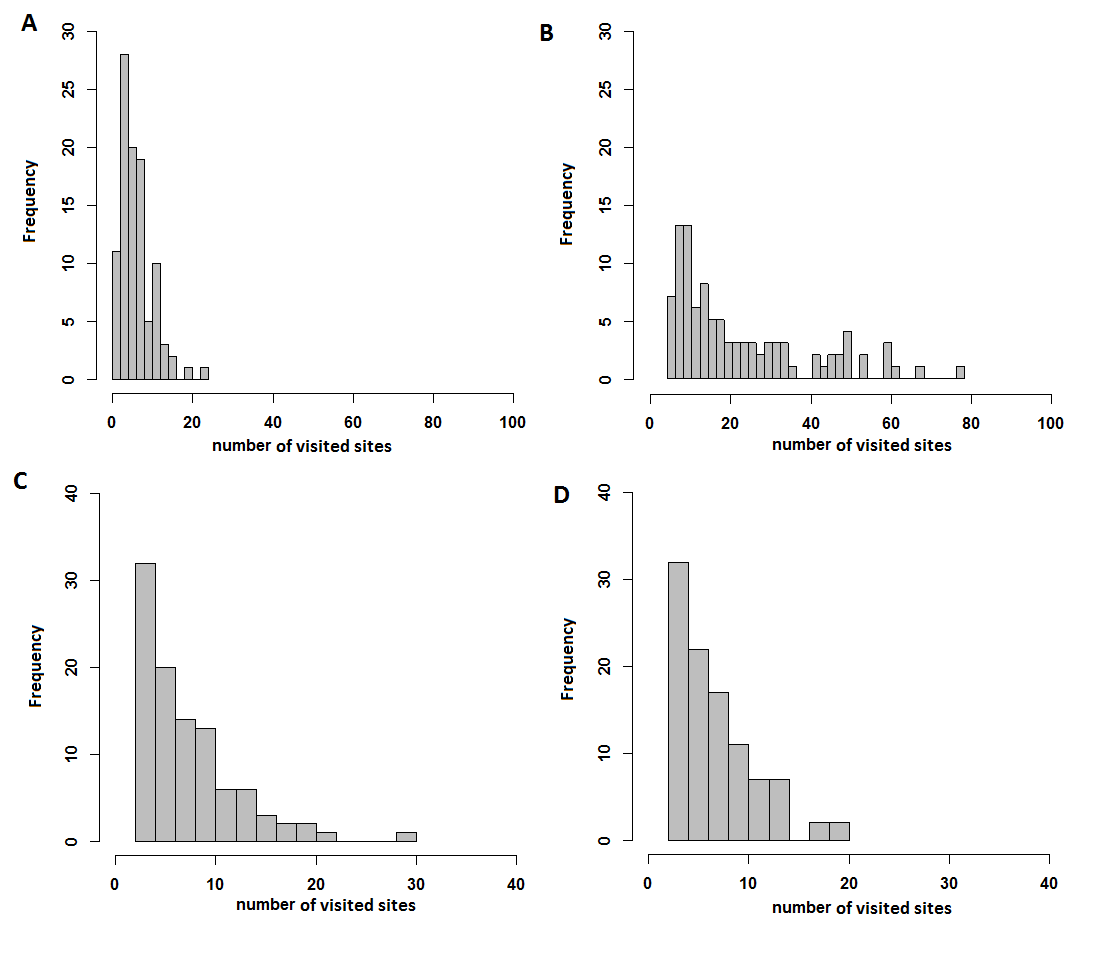


Figure S3


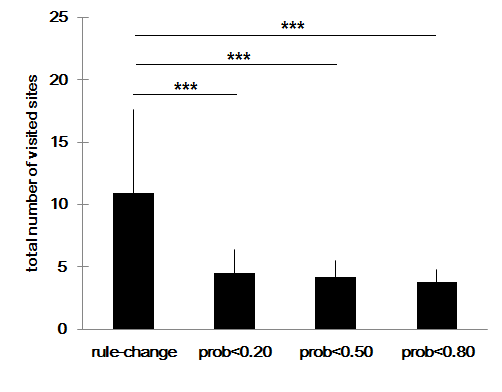


Figure S4

Figure S1. Examples for density plots of agent’s trajectories. A. Non-shift version for the center of home range regions. B. Shift version for the center of home range regions. The black cross indicates start position of the agent.

Figure S2. Distributions of the number of re-visit for each site and mean squared displacements in random-choice algorithm. A. The relationship between the number of re-visit for each site and that of cumulative distribution. B. The relationship between mean squared displacements <*R*^2^> and squared time *t*^2^ from 100 trials.

Figure S3. Distributions of the number of visited sites after each trial obtained from 100 trials in various parameter changes. A. Rule-change model with the number of distributed sites = 500. B. Rule-change model with the number of distributed sites = 1,500. C. Rule-change model with θ=10.00. D. Rule-change model with θ=20.00.

Figure S4. Averaged total number of visited sites obtained from 100 trials in rule-change model and spatial memory-based model with various parameters (*prob* = 0.20, 0.50, 0.80). Black bars indicate error bars. ^***^*P*<0.001.
